# Large-scale adverse effects related to treatment evidence standardization (LAERTES): an open scalable system for linking pharmacovigilance evidence sources with clinical data

**DOI:** 10.1186/s13326-017-0115-3

**Published:** 2017-03-07

**Authors:** The Knowledge Base workgroup of the Observational Health Data Sciences and Informatics (OHDSI) collaborative, Richard D. Boyce, Erica A. Voss, Vojtech Huser, Lee Evans, Christian Reich, Jon D. Duke, Nicholas P. Tatonetti, Tal Lorberbaum, Michel Dumontier, Manfred Hauben, Magnus Wallberg, Lili Peng, Sara Dempster, Yongqun He, Anthony G. Sena, Vassilis Koutkias, Pantelis Natsiavas, Patrick B. Ryan

**Affiliations:** Suite 419, 5607 Baum Blvd, Pittsburgh, PA 15202 USA

**Keywords:** Pharmacovigilance, Post-market drug safety, Clinical terminologies, Linked-data

## Abstract

**Background:**

Integrating multiple sources of pharmacovigilance evidence has the potential to advance the science of safety signal detection and evaluation. In this regard, there is a need for more research on how to integrate multiple disparate evidence sources while making the evidence computable from a knowledge representation perspective (i.e., semantic enrichment). Existing frameworks suggest well-promising outcomes for such integration but employ a rather limited number of sources. In particular, none have been specifically designed to support both regulatory and clinical use cases, nor have any been designed to add new resources and use cases through an open architecture. This paper discusses the architecture and functionality of a system called Large-scale Adverse Effects Related to Treatment Evidence Standardization (LAERTES) that aims to address these shortcomings.

**Results:**

LAERTES provides a standardized, open, and scalable architecture for linking evidence sources relevant to the association of drugs with health outcomes of interest (HOIs). Standard terminologies are used to represent different entities. For example, drugs and HOIs are represented in RxNorm and Systematized Nomenclature of Medicine -- Clinical Terms respectively. At the time of this writing, six evidence sources have been loaded into the LAERTES evidence base and are accessible through prototype evidence exploration user interface and a set of Web application programming interface services. This system operates within a larger software stack provided by the Observational Health Data Sciences and Informatics clinical research framework, including the relational Common Data Model for observational patient data created by the Observational Medical Outcomes Partnership. Elements of the Linked Data paradigm facilitate the systematic and scalable integration of relevant evidence sources.

**Conclusions:**

The prototype LAERTES system provides useful functionality while creating opportunities for further research. Future work will involve improving the method for normalizing drug and HOI concepts across the integrated sources, aggregated evidence at different levels of a hierarchy of HOI concepts, and developing more advanced user interface for drug-HOI investigations.

## Background

A recent report from the United States Department of Health and Human Services noted that, while medications help millions of people live longer and healthier lives, they are also the cause of approximately 280,000 hospital admissions each year and an estimated one-third of all hospital adverse events [1]. The field of post-market drug safety surveillance focuses on applying the most current methodological advances to help identify undesired effects of drugs and biologics. One of the major opportunities and challenges to safety investigators is that there are many disparate evidence sources from which a safety concern might either be identified or evaluated. These may include spontaneous reporting systems, electronic health records, the literature, Web search logs, and social media. [2–7]. Safety concerns can also be predicted from the knowledge about the chemical structure and pharmacological properties of drugs [8]. Combining multiple sources of biomedical evidence has been shown to have value for improving the precision of automated signal identification [9], and for identifying both established [10] and new safety concerns [11].

Consistent with these results, there has been a recent call for more research on “combinatorial signal detection” that is defined as integrating multiple disparate evidence sources while making the evidence computable from a knowledge representation perspective (i.e., semantic enrichment) [12]. Examples of such features include:

- The use of formal (i.e., logically defined and computable) definitions for the meaning of entities represented in the database such as drugs and HOIs.
- Formally defined relationships between the entities represented in each integrated evidence source.
- Computational methods for inferring new knowledge from evidence, for example using rule-based or machine learning methods.

**Fig. 1:**
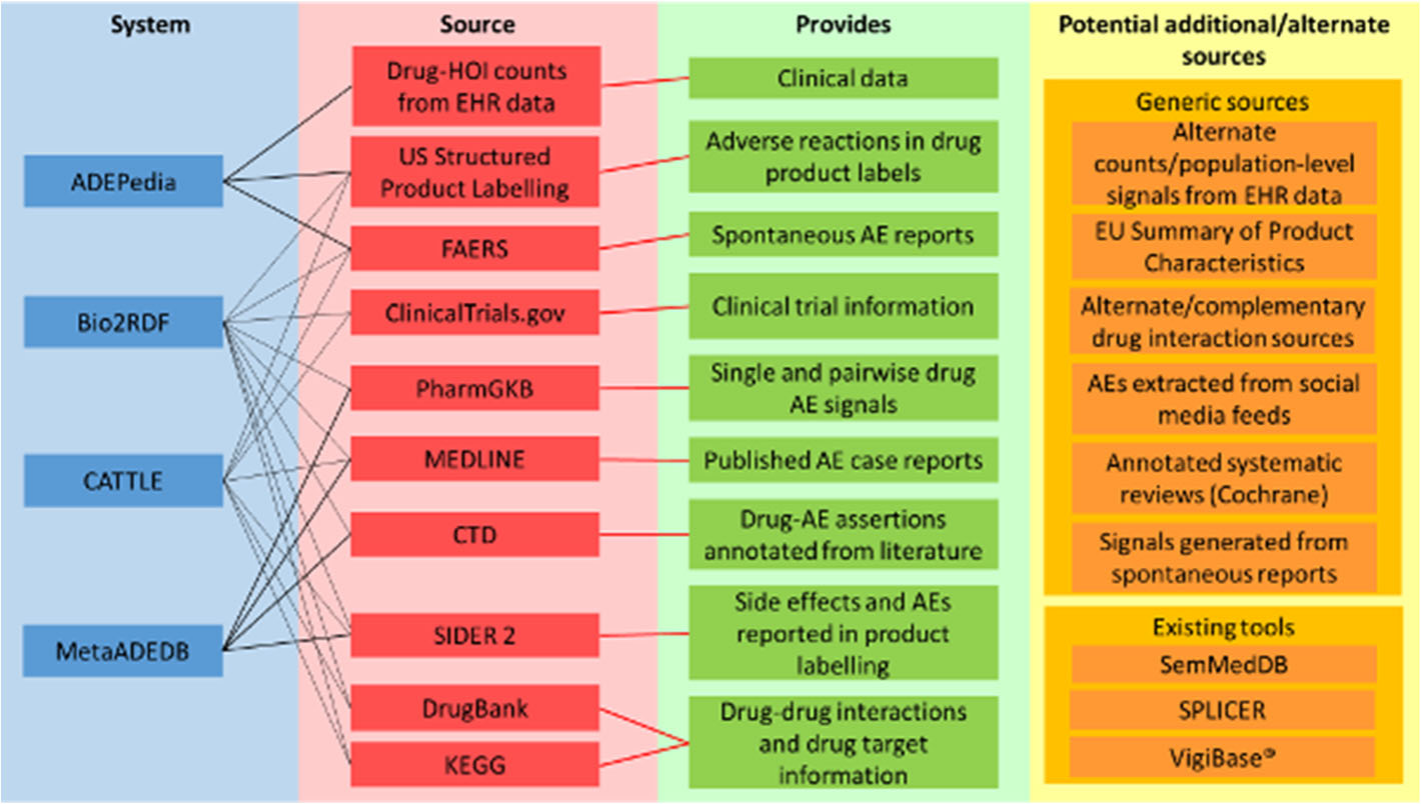
The information sources of existing knowledge-based systems for pharmacovigilance. Citations to the sources mentioned can be found in the “Background” section. EHR: electronic health record, AE: adverse event, EU: European Union, FAERS: Food and Drug Administration Adverse Event Reporting System, CTD: Comparative Toxicogenomics Database

Existing frameworks that have integrated various sources in a way that provide some of these features include ADEPedia [13, 14], MetaADEDB [15], CATTLE [16], and Bio2RDF [17]. Fig. 1 shows the evidence sources integrated into these systems. As the figure indicates, there are several alternate sources that could be integrated including VigiBase®, pharmacovigilance signals from multiple sources (or using alternative methods) [18], electronic health records signals from multiple sources (or using alternative methods) [19], alternate approaches to extracting safety concerns from unstructured text using natural language processing [20], and various sources of drug-drug interaction evidence [21].

With the exception of Bio2RDF, none of the aforementioned systems have an open architecture that would enable the integration at large-scale, systematically, while facilitating the integration of new sources. Bio2rdf does have an open architecture. The code for loading a data source into Bio2RDF is open source enabling motivated scientists to create a local version of Bio2RDF and then integrate a new source by writing code that translates the sources data into a Resource Description Framework (RDF) graph according to Bio2RDF conventions [22]. They can also edit the translation code for existing sources to alter decisions that are made during the integration process. This ability is important, because there are a variety of decisions made on how these sources are integrated that can influence downstream analyses. Table 1 shows some of the decisions to be made with respect the general Extract, Translate, and Load (ETL) process for pharmacovigilance (i.e., not specific only to Bio2RDF).

**Table 1:** Decisions that are made during the process of integrating sources that can influence downstream pharmacovigilance analyses

Data Type	Feature	Option for variability	Performance questions
Product labels	Product label outcome mention	Named entity performance (PPV and sensitivity)	Do improvements in entity recognition performance improve system recall and precision?
		Section location (e.g., anywhere vs specific sections)	Does identifying which sections are more informative than others reduce noise?
	Frequency information	Threshold variation	Does incorporation of ADE frequency improve performance? What cut-off should be used?
Pharmacovigilance DBs (e.g. FAERS, MedEffect, VigiBase)	Minimum detectable relative risk	Threshold variation	What is the appropriate cut-off for MDRR? Is it HOI specific?
		Database (s) chosen	Does the database influence the value of MDRR for this task?
	Risk identification method	Disproportionality metric	What metric (e.g. PRR, EBGM, IC) leads to the best performance? Is it HOI specific?
	Number of cases in FAERS	Threshold variation	What is the appropriate cut-off for number of case reports?
Drug Indication DB	Indication listings in FDB	Yes/no and when mentioned	Does using on-label and off-label indication knowledge improve performance?
Indexed literature	Number of relevant publications from the indexed literature	Threshold variation	Is there an appropriate cut-off for number of publications? What is its variability relative to specific HOIs and drugs?
	Source of relevant publications from the indexed literature	Varying the combination of sources	Should we be selective about the sources used or chose all of them?
	Drug and outcome mention in relevant indexed literature	Named entity performance	Do improvements in entity recognition performance improve system recall and precision?
		Main MeSH terms vs supplemental	What is the value of MeSH supplemental terms relative to the primary index terms?
		Scientific discourse tag of the location of mention (e.g., intro, methods, results, conclusions)	Does limiting identification of drug-HOI co-mention to specifically tagged text excerpts improve performance?
		Publication type label (randomized trial, case report, etc.)	Should the publication type of the drug-HOI co-mention be tracked and possibly weighted to improve performance?
		Source of publication type label (Embase, MeSH)	Is one publication type indexing system better than the other for the question answering task, or should they be combined?
		Topic of the source publication based on latent semantic indexing	Does the use of tags assigned to text sources by latent semantic indexing improve system performance if used as a feature?
Observational health data (claims + EHR)	Minimum detectable relative risk	Threshold variation	What is the appropriate cut-off for MDRR? Is it HOI specific?
		Database (s) chosen	Does the database influence the value of MDRR for this task?
	Risk identification method	Analytic method	What method (e.g. disproportionality analysis, self-controlled case series, IC temporal pattern discovery, high-dimensional propensity score) leads to the best performance? Is it HOI specific?
	Cohort selection	Patient ethnicity, age, sex, co-morbidities, concurrent medications	Does cohort selection using these features affect model performance? What is the appropriate size and diversity of the cohort to reduce noise and bias?
	Drug exposure conditions	Length of exposure, dosage	Does selecting minimum exposure duration criteria and/ or drug dosage information improve performance?
	Study replicability	Number of locations for confirming results	How many replicates of the study should be performed at different institutions?
	Observation period	Observation duration threshold	Does setting minimum observation period durations improve performance?

*PPV*: positive predictive value, *OMOP*: Observational Medical Outcomes Partnership, *ADE*: adverse drug event, *MDRR*: minimal detectable reporting ratio, *HOI*: health outcome of interest, *DB*: database, *FAERS*: Food and Drug Administration Adverse Event Reporting System, *EBGM*: empirical Bayes geometric mean. *IC*: information component, *FDB*: First Data Bank (commercial drug knowledge base), *EHR*: electronic health record

In a previous paper [23], we presented the vision of establishing an open-source community effort to develop a global knowledge base of known associations between drugs and HOIs: one that brings together and standardizes all available information for all drugs and all HOIs from all electronic sources pertinent to drug safety. To make this vision a reality, a workgroup within the Observational Health Data Sciences and Informatics (OHDSI) collaborative [24] was organized for the purpose of developing a standardized knowledge base for the effects of medical products, and an efficient procedure for maintaining and expanding it. The main purpose of the knowledge base is to make it simpler to access, retrieve, and synthesize evidence so that users can develop an assessment of causal relationships between a given drug and HOI as accurate as current evidence provides.

This paper discusses the results of this workgroup thus far. Specifically, the paper discusses the architecture and functionality of a prototype system called Large-scale Adverse Effects Related to Treatment Evidence Standardization (LAERTES). LAERTES provides open and scalable architecture for linking evidence sources relevant to investigating the association of drugs with HOIs. The remaining sections of this paper will discuss the motivating user story, the system’s architecture, implementation details, and the current beta release.

## Implementation

### Motivating user story and goal

Safety physicians and risk management analysts investigate new adverse drug event reports and emerging drug safety signals. A typical way to express the requirements imposed on a software system by a specific user group is via the so-called “user stories”. The user story which drove the development of the LAERTES platform is defined as follows:

“As a safety physician or risk management analyst monitoring the safety of a marketed drug, I want to do a comprehensive search across known or emerging drug-HOI evidence so I can thoroughly and expeditiously triage emerging potential safety signals and assess their potential impact.”

In order to do that, a number of tasks have to be carried out including:

1. quickly determining if a specific adverse event has been previously reported for a given drug;
2. gauging if a potential safety concern is at the clinical drug (e.g. ‘simvastatin 20 mg oral tablet’), ingredient (e.g., ‘simvastatin’), or class (e.g., ‘statins’) level;
3. assessing the credibility of the sources reporting the association, and
4. deciding what priority an adverse event signal might warrant for further investigation.

### LAERTES system and data architecture

**Fig. 2:**
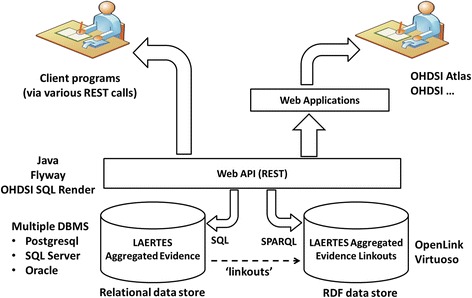
The overall architecture of LAERTES within the OHDSI clinical research software environment. REST: representational state transfer, OHDSI: Observational Health Data Sciences and Informatics, API: application programming interface, DBMS: database management system, CDM: common data model, OA: Open Annotation Data, RDF: Resource Description Framework

The LAERTES system architecture (Fig. 2) includes three main components that operate within a larger software ecosystem provided by the OHDSI clinical research framework. The three components are 1) a Resource Description Framework (RDF) data store that represents all included evidence sources as Open Annotation Data (OA) model [25], 2) a relational data store that enables both summary queries providing an overview of evidence across all included sources, and drill down queries that examine important information on specific evidence items; and 3) a web services layer that hides the details of how to query the RDF and relational component so that client programs can more easily benefit from their combined functionality. The next few sub-sections discuss these components in more detail.

### RDF data and the “drill down” use case

RDF is a standard developed through the World Wide Web Consortium (W3C) that uses Uniform Resource Identifiers (URIs) and a graph-based data model to represent any kind of connected data [26]. Since the introduction of RDF as a key component of the Semantic Web, the standard has become widely used, especially in the biomedical sciences [27]. In comparison with the relational data model, the underlying graph model of RDF makes querying across heterogeneous data sets simple. The data represented in RDF data are computable and semantically non-ambiguous through the use of URIs and ontologies. RDF “Linked Data” provides a convention to ensure that all data items across multiple connected graphs are easily accessible using standard web technology.

In LAERTES, a specific piece of evidence in favor or against an association between a drug and an HOI from any integrated data source is represented using the Open Annotation Data (OA) model. OA is a standard for representing human and computer annotations that is gaining broad adoption among many publishing communities. In LAERTES, every item of evidence about a drug-HOI pair is represented as an OA resource present in the RDF data store (Fig. 2). The reasons for this approach are to 1) use a single standard approach to representing evidence items regardless of the source and 2) harness the aforementioned benefits of Linked Data.

**Fig. 3:**
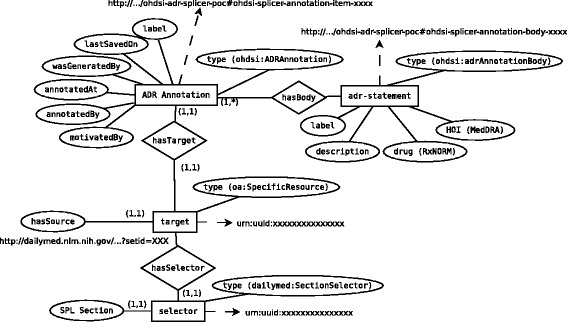
An entity relationship diagram showing how data from US product labeling is represented as a semantically enriched Open Annotation Data graph

Each OA resource provides the data about the source of a specific evidence item (the “target”) and the semantic tags used to identify the record as a relevant evidence item (the “body” or “bodies”). The body of each OA has resources for drug, drug group, and HOI concepts that are represented using standard vocabularies (Medical Subject Headings (MeSH), Medical Dictionary for Regulatory Activities (MedDRA), Systematized Nomenclature of Medicine - Clinical Terms (SNOMED-CT), and RxNorm). To enable integration with other data sources in the OHDSI clinical research framework and facilitate the reuse of the LAERTES platform in the context of the OHDSI analytical tools, the source codes from the relevant terminology are replaced with the equivalent concept_id from the concept table which is part of the Observational Medical Outcomes Partnership (OMOP) Standard Vocabulary [28]. Fig. 3 shows an entity relationship diagram for the OA resources created for adverse drug reactions extracted from US drug product labeling. Graphs with the same basic structure (an annotation resource linked to a target and a body) are created for other evidence sources but given a different type and selectors that are appropriate to the source. For example, an OA resource that represents a drug-HOI evidence from MEDLINE MeSH tag assignment would be given the type ohdsi:PubMedDrugHOIAnnotation and a selector with the exact text of the title and abstract.

### Relational data and the “summary” use case

As the system diagram in Fig. 2 shows, aggregated evidence exists for LAERTES in a relational data store. Within the data store, there are “linkouts” to OA resources (described below). A web application programming interface (API) is able to interact with both the relational data store as well as the RDF linkouts. This interoperable representational state transfer (REST) API can be leveraged for user interaction either directly or via third-party applications.

**Fig. 4:**
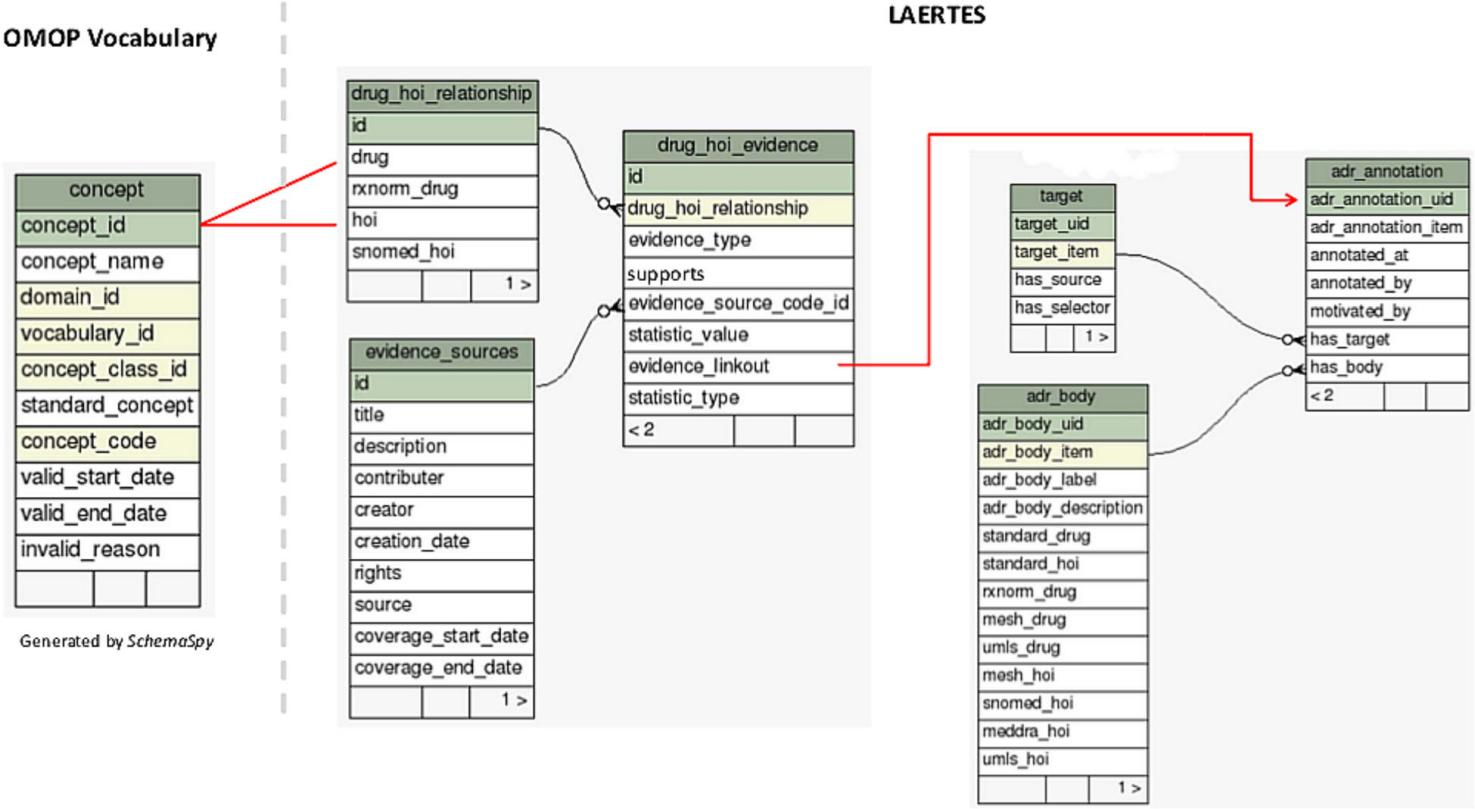
The data architecture of LAERTES. The system leverages the OMOP Vocabularies to describe drugs and health outcomes of interest via standardized vocabulary concepts (concept table). LAERTES stores aggregated evidence in a summary table (drug_hoi_evidence) that provides a “linkout” (evidence_linkout) to an Open Annotation Data representation of the source data. In the relational database, the linkout functions as a foreign key to the adr_annotation table through a table (not shown) that maps the linkout to annotation identifiers (adr_annotation_uid). Client programs can also use the linkout as a URL to retrieve JSON data from an RDF store that has a linked data version of the source open annotation data

The schema for the primary tables used in the relational data store is shown in Fig. 4. The evidence_sources table holds metadata on each data source that has been loaded into LAERTES. The table drug_hoi_relationship is used to hold the concept identifiers for the drug and HOI pairs used in the drug_hoi_evidence table. Drug and HOI concepts in this table have been converted from the source terminologies (e.g., MeSH, MedDRA) to RxNorm and SNOMED-CT concepts using relationships present in the Standard Vocabulary provided by the OHDSI clinical research framework (“OMOP Vocabulary” in Fig. 4). Natural language processing (NLP) is applied to sources that do not use a specific terminology. For example, the validated NLP tool SPLICER [29] is used to process United States product labeling from unstructured text to RxNorm drug and MedDRA HOI mentions. A key point is that clinical datasets represented in the OHDSI clinical research framework will use the standardized concepts from RxNorm and SNOMED making it possible to create queries that join the merged evidence in LAERTES with clinical data.

The drug_hoi_evidence table provides aggregate summary statistics for every drug-HOI pair from a given source, noting, wherever possible, if the evidence supports or refutes an association. Aggregation across the sources is possible because all drug and HOI concepts are translated to RxNorm and SNOMED-CT, respectively. The aggregation is based on a number of different scores and coefficients. For example, a specific drug-HOI association might have evidence from spontaneous reporting in the form of adverse event counts as well as the results of disproportionality analyses over the reporting database (i.e., proportional reporting rates and other signal statistics [30]). If so, the drug_hoi_evidence table would hold a distinct record for both statistics while indicating each record’s type in the statistic_value field.

### Evidence “linkouts” – the bridge between the summary and “drill down” use cases

An important data element in the drug_hoi_evidence table is the evidence_linkout column. This holds a URL that functions as a foreign key to the adr_annotation table through a table (not shown) that maps the linkout to annotation identifiers. This enables analysts using the relational database to examine an OA represention of the source records used to create the summary data located in drug_hoi_evidence. Client programs can also use the linkout as a URL to retrieve JSON data from the RDF store that has a linked source open annotation data for the purpose of displaying this evidence. An example will help clarify the functionality. First, the following SPARQL script (executable on the public OHDSI RDF store [31]) shows how to query for a source document in the RDF store shown in the system diagram (Fig. 2):

- # The URI to the source document from which the anonymous PubMed drug-HOI OA
- # resource represented by ?s is returned in the ?sourceDocument variable
- PREFIX oa: < http://www.w3.org/ns/oa #>
- PREFIX ohdsi: < http://purl.org/net/ohdsi #>
- SELECT ?sourceDocument
- WHERE {
- ?s a ohdsi:PubMedDrugHOIAnnotation;
- oa:hasTarget ?target.
- ?target oa:hasSource ?sourceDocument.
- } LIMIT 10

This query can be used to retrieve the evidence item for the specific drug-HOI pair “Simvastatin 20 MG Oral Tablet” (identifier:1539411) and HOI “muscle weakness” (identifier: 36516876) from a specific source (in this case the a MEDLINE record). The important changes are shown in bold font:

- # The URI to the source document that provides evidence for an association between simvastatin
- # and Rhabdomyolysis is returned in the ?sourceDocument variable
- PREFIX oa: < http://www.w3.org/ns/oa #>
- PREFIX ohdsi: < http://purl.org/net/ohdsi #>
- SELECT ?sourceDocument
- WHERE {
- ?s a ohdsi:PubMedDrugHOIAnnotation;
- oa:hasTarget ?target;
- oa:hasBody ?body.
- **# “simvastatin”**
- **?body ohdsi:ImedsDrug ohdsi: 1539403.**
- **# “Rhabdomyolysis”**
- **?body ohdsi:ImedsHoi ohdsi: 45619309.**
- ?target oa:hasSource ?sourceDocument.
- }

SPARQL queries like this one can be sent to an RDF endpoint, in order to facilitate the reuse of the annotations through produced other Linked Data applications. Returning our focus to the relational data model (Fig. 4), each of the entries in the evidence_linkout column holds a URL that encodes the specific SPARQL query needed to retrieve OA resources for a given drug, HOI, and evidence source. Testing revealed that the needs and preferences of various users required the ability to access the open annotation data as either RDF or relational data (for example, a pharmaceutical company’s IT infrastructure might be more amenable to working with relational data rather than RDF). At the same time, other users more familiar with the interoperability and inference strengths of RDF Linked Data will benefit from the RDF representation. To accommodate this dual functionality, the exact same encoded URLs are used as foreign keys to the adr_annotation table through another table that maps the linkout to annotation identifiers. The target and adr_body tables hold a copy of the OA target and hasBody data (Fig. 3).

### Evidence “rollups”

**Fig. 5:**
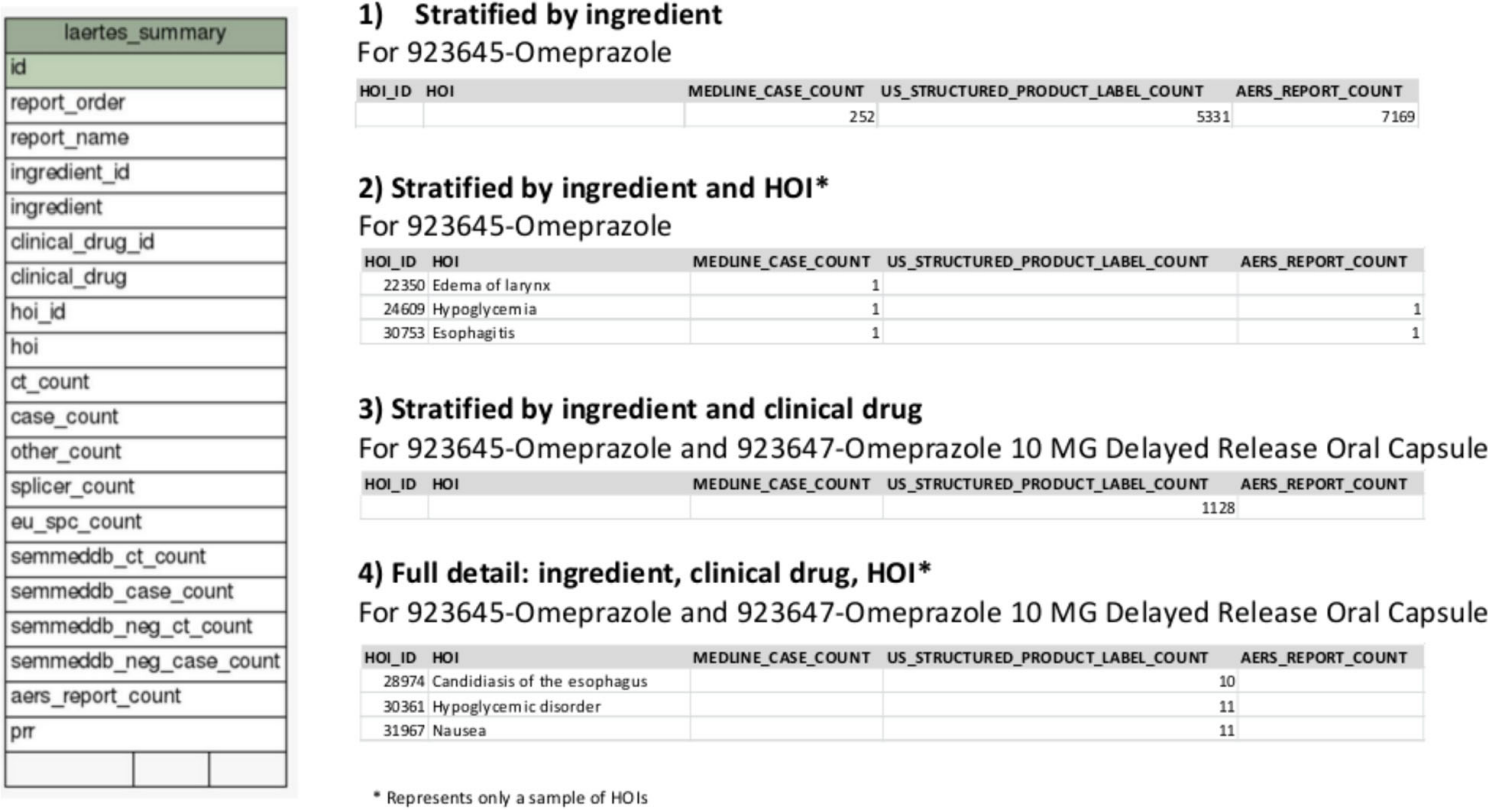
The drug “roll-up” table and example reports by order identifier

One of the key features of the proposed platform is that safety evidence may be linked to the drug at different levels of granularity: at the clinical drug product level, comprising the active ingredients, strength, formulation, and brand name for the product, but may also be more coarsely defined simply as evidence for a particular active ingredient. For example, an adverse event might be mentioned in the drug product label for only one clinical drug containing a specific active ingredient. However, a published case report might discuss an adverse event that appears to be associated with all the drugs containing the active ingredient. LAERTES supports querying the evidence at four different “rollup” levels: (1) by RxNorm drug ingredient, (2) by RxNorm drug ingredient and SNOMED-CT HOI, (3) by RxNorm drug ingredient and RxNorm clinical drug, and (4) by full detail which was across the RxNorm drug ingredient, RxNorm clinical drug, and SNOMED_CT HOI. These “rollup” queries are supported by a table called laertes_summary (Fig. 5). Data are aggregated from the evidence items and inserted into this table during the evidence load process using queries against the tables shown in Fig. 4.

## Results

### Technical implementation

At the time of this writing, six evidence sources have been loaded into LAERTES representing three literature sources, two drug product label sources, and one spontaneous reporting source. Table 2 provides a brief summary of each source, the methods used to normalize drug and HOIs to RxNorm and SNOMED-CT respectively, and the number of drug-HOI pairs that were available before and after mapping. The specific code used to perform normalization is available from the project’s GitHub site [32]. In general, custom Python scripts execute queries that identify OHDSI concept identifiers for the source drug and HOI concepts, and then use OHDSI Standard Vocabulary mappings to translate from source concepts to RxNorm and SNOMED-CT. Table 3 provides the overlap of distinct drug-HOI pairs at the drug ingredient level across the three broad categories of evidence (drug product labeling, published literature, and spontaneous reporting).

**Table 2:** Distinct drug-Health Outcome of Interest pairs by source

Source description	Drug and HOI mapping method	Distinct drug-HOI pairs in source	Distinct drug-HOI pairs in LAERTES (%)
Adverse drug reactions mined from US drug product labels using a validated natural language processing tool called SPLICER [29]	Drugs were coded using RxNorm and HOIs using MedDRA. The OMOP Standard Vocabulary was used to map MedDRA to SNOMED-CT.	272 436^a^	254 738 (93%)
Adverse drug events extracted from EU Summary of Product Characteristics by the PROTECT project	Drugs were mentioned by name and HOIs using MedDRA codes. Drug names were mapped to RxNorm using a combination of simple string matching and Bioportal ontology searches. Many combination products and some individual drugs were not mappable. All mappings were manually reviewed for accuracy.	26 989	24 537 (91%)
FDA Adverse Event Reporting System counts and Proportional Reporting Ratio from [45]	The OHDSI Usagi tool [46] was used to map drug and HOI mentions to RxNorm and MedDRA. The OMOP Standard Vocabulary was used to map MedDRA coded HOIs to SNOMED-CT. A paper describing the database and mapping method has been published [47].	3 766 382	2 753 078 (73%)
Abstracts from titles and abstracts indexed in MEDLINE that describe drug-HOI evidence according to MeSH indexing [48]	Drug and HOI concepts were both coded using MeSH. The OMOP Standard Vocabulary was used to map from MeSH drug concepts to RxNorm and MeSH HOI concepts to SNOMED-CT.	79 119^b^	77 395 (97.8%)
Sentence spans from titles and abstracts indexed in MEDLINE that describe drug-HOI evidence according to queries against the Semantic Medline database	Drug and HOI concepts were both coded using UMLS concept identifiers. The UMLS Metathesaurus MRCONSO table was used to map concepts to RxNorm, MeSH, MedDRA, and SNOMED-CT. The OMOP standard vocabulary was then used to map drug concepts only available as MeSH to RxNorm and HOI concepts only available as MedDRA or MeSH concepts to SNOMED-CT.	5 023^b^	2 813 (56%)
Chemical disease associations from the Comparative Toxicogenomics Database	Drug and HOI concepts were both coded using MeSH. The OMOP Standard Vocabulary was used to map from MeSH drug concepts to RxNorm and MeSH HOI concepts to SNOMED-CT.	503 835	432 850 (86%)

^a^SPLICER drug-hoi pairs are at the clinical drug level. All other sources are at the ingredient level. ^b^Does not include drug-HOI evidence where the source refers to the drug by its MeSH pharmacologic group name. *EU*: European Union, *FDA*: Food and Drug Administration, *HOI*: Health outcome of Interest, *OMOP*: Observational Medical Outcomes Partnership, *US*: United States, *MedDRA*: Medical Dictionary for Regulatory Activities, *MeSH*: Medical Subject Headings

**Table 3:** Overlap of distinct drug-HOI pairs at the drug ingredient level after mapping drugs to RxNorm and HOIs to SNOMED-CT

Literature (MEDLINE and CTD) vs spontaneous reporting (*n* = 3 049 743)	Product labeling (US and EU) vs spontaneous reporting (*n* = 2 702 577)	Literature (MEDLINE and CTD) vs product labeling (US and EU) (*n* = 566 379)	All three (*n* = 3 057 406)
119 293 (3.9%)	87 279 (3.2%)	14 838 (2.6%)	14 295 (0.5%)

The counts and percentages shown contrast the sum of the union (shown in the heading) and intersection of the distinct drug-HOI pairs from both sources mentioned. CTD: Comparative Toxicogenomics Database, EU: European Union, US: United States

Each evidence source was processed using a custom Extract, Translate, and Load (ETL) module developed in Python. All ETL modules follow a similar pattern involving 1) transforming the source data to an RDF OA graph and 2) loading the graph into an RDF endpoint, and 3) executing a query that generates statistics (e.g. count data) and linkouts. Each linkout is URL-encoded and then shortened using a custom implementation of the HarryJerry Linx URL shortener [33]. Python scripts merge the count and linkout data from each source into data files that are loaded into the relational database. All of the code used to create the current implementation is available from the open source OHDSI/KnowlegeBase project [32]. Evidence source updates currently occur every 3 to 6 months and follow the same workflow.

### Accessing and using data in LAERTES

**Fig. 6:**
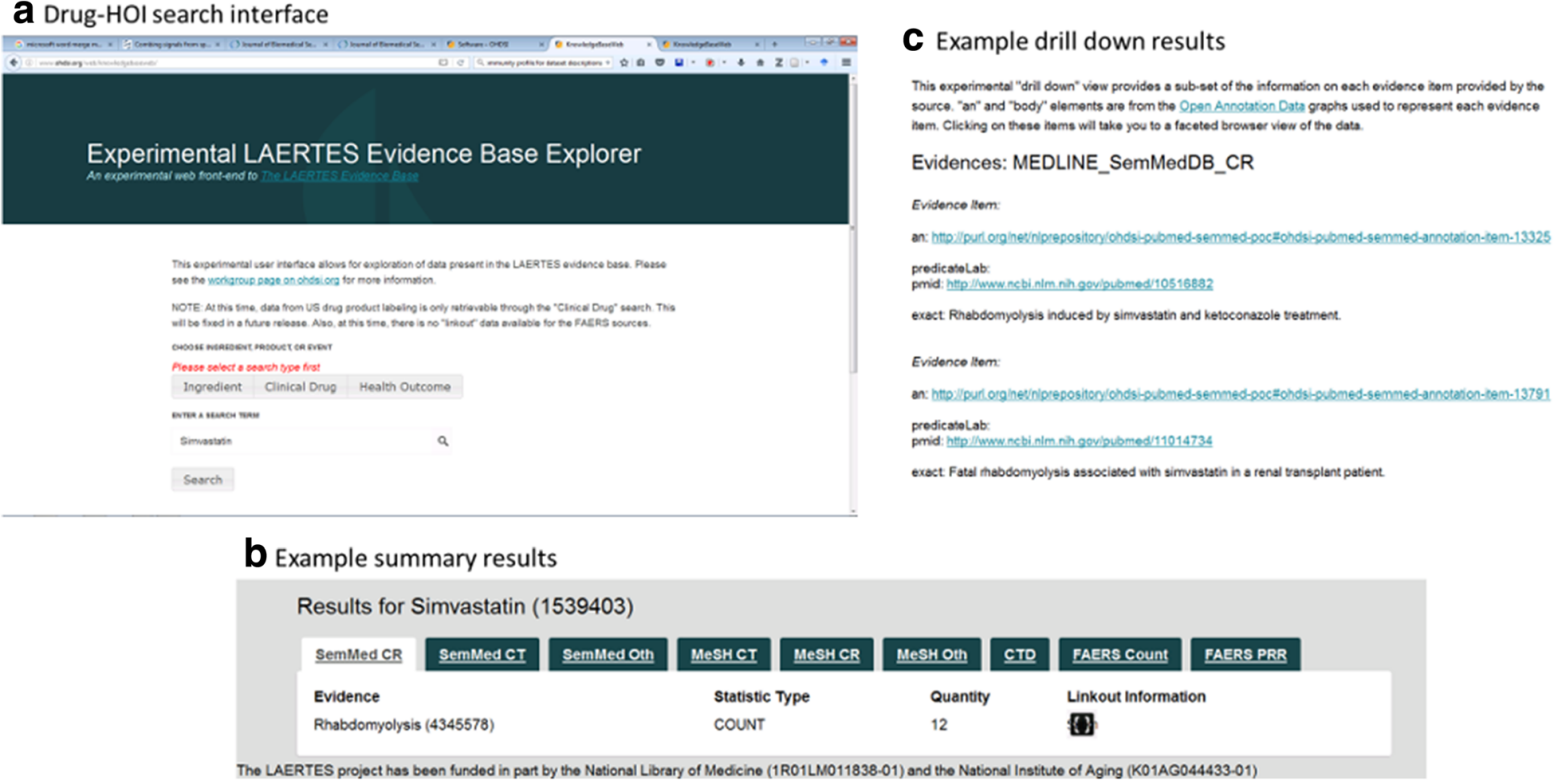
Experimental user interface to the LAERTES evidence base

Interested persons can currently access the data in LAERTES in a few different ways. The RDF database is hosted on a public-facing server [31] and the authors can provide direct access to the relational database upon request (e.g., via direct email or a request posted to forums.ohdsi.org). A proof-of-concept user interface has been developed [34] and is also hosted on a publicly accessible server (Fig. 6) [35]. This simple user interface allows users to query LAERTES using OMOP concept identifiers or concept names for drug ingredients, drug products, or HOIs. The system presents a summary of query results in a simple tabular format. Links are provided so that users explore “drill down” information. This system uses some of the several REST API calls that are documented on the OHDSI Wiki [36].

Progress has been made on integrating the cross-platform web services provided by the current version of LAERTES with other applications in the OHDSI clinical research framework. Specifically, the vocabulary browsing component of the OHDSI ATLAS web based tool [37] can use the LAERTES API to retrieve the available evidence of drug-HOI associations that it displays to users. Furthermore, a new extension to ATLAS is under development that will enable searching for drugs-HOI pairs with no evidence in any included source. The outputs of this program are called “negative controls” and can be used for investigating drug-HOI associations using observational data to “calibrate” the confidence intervals of statistical estimates to address hidden biases within the observational dataset [38].

### How LAERTES can support the user scenario

The current version of LAERTES is a prototype that can support some of the requirements of the safety physician and risk management analyst whose user story is mentioned at the beginning of this paper:

1. Quickly determining if a specific adverse event has been previously been reported for a given drug: LAERTES currently brings together three main types of information where drug-HOI associations are reported (spontaneous reports, labeling, and published literature). The system’s open architecture makes it possible to add additional sources such as data from clinical trials. Because drugs and HOIs are normalized from the source terminologies to RxNorm and SNOMED-CT, a single query accomplishes the task of identifying existing evidence from any of the included sources. This is possible via SPARQL and SQL queries as well as through the Web API.
2. Identifying if a potential safety concern is at the clinical drug, ingredient or class level: LAERTES allows searches specifically at the clinical drug or ingredient levels while also providing evidence “rollups” which aggregate evidence at the ingredient level (see Section *Evidence “Rollups”*). However, there currently is only one source integrated into LAERTES that provides evidence at the clinical drug level (US drug product labeling). This is not likely to be a limitation since, in the OHDSI clinical research framework, all clinical drug data is loaded into the CDM drug_exposure table (not shown) and then also represented at the ingredient level in the CDM drug_era table (also, not shown).
3. Identifying the credibility of the sources reporting the association: Both the relational and RDF components of LAERTES explicitly note the source of an evidence item and, if relevant, the particular type of evidence. For example, an evidence item from a literature source would be explicitly tagged with the method used to identify the evidence (MeSH tags or natural language processing) and the study type of the article from which the item was found (clinical trial, case report, or other). Similarly, drug-HOI evidence from product labeling is tagged with the specific method used to identify it and the section from which it was pulled. These tags are intended to be useful for filtering or prioritizing evidence based on a user’s perception of the relative credibility of the sources or evidence type. However, further research is necessary to test this assumption and identify the requirements for other ways to help users more rapidly assess evidence credibility.
4. Deciding what priority an adverse event signal might warrant for further investigation: At present, LAERTES provides only an experimental graphical user interface (Fig. 6) but the workgroup is actively working on a new user interface that will fully support prioritizing an adverse event signal for further investigation. The new user interface is being designed to help users take full advantage of the new possibilities created by bringing together the multiple sources of drug-HOI evidence into the OHDSI clinical research framework. As Listing 1 shows, LAERTES is designed to work seamlessly with patient data that has been loaded into the OMOP CDM. As a result, users would be able to directly generate new drug-HOI evidence from one or more clinical datasets (claims, electronic health records, or registries) using OHDSI population-level effect estimation methods [39]. These methods, which are in development, promise rapid large-scale exploration of a suspected drug-HOI association using causal considerations which include strength of association, consistency, temporality, experiment, plausibility, coherence, biologic gradient, specificity, and analogy [40].

#### Listing 1

An example of querying patient data on the OHDSI CDM using drug HOI pairs present in the LAERTES evidence base. The query counts the number of cases present in the clinical dataset where a patient condition is recorded within 30 days of the start of a drug (as indicated by data in the CDM drug_era table). The results shown are a subset of the results that were generated when the query was ran on a simulated population available to the OHDSI research community and the general public [41]. The results provide summary information and Web links that point to a summary of each source evidence item in the LAERTES RDF store. These links could be used by a third-party application to help the user further “drill down” into the evidence that associated the drug with the HOI.

-- retrieve the count of distinct patients exposed drug-HOI combination for which there is
-- evidence in LAERTES from MEDLINE or European product labeling using RxNorm drug identifier,
-- SNOMED-CT drug identifier, evidence type, evidence ‘linkout’, and
- select rxnorm_drug, snomed_hoi, evidence_type, evidence_linkout,
- count(distinct person_id) pcount
- from
- (select sub1.person_id,
- drug_hoi_relationship.rxnorm_drug,
- sub1.drug_era_start_date,
- sub1.drug_era_end_date,
- drug_hoi_relationship.hoi,
- drug_hoi_relationship.snomed_hoi,
- sub1.condition_era_start_date,
- drug_hoi_evidence.evidence_type,
- drug_hoi_evidence.evidence_linkout
- from
- drug_hoi_evidence inner join drug_hoi_relationship
- on drug_hoi_evidence.drug_hoi_relationship = drug_hoi_relationship.id
- inner join
- (select drug_era.person_id,
- drug_era_start_date,
- drug_era_end_date,
- drug_concept_id,
- condition_era_start_date,
- condition_concept_id
- from drug_era
- inner join condition_era on drug_era.person_id = condition_era.person_id
- where condition_era.condition_era_start_date > drug_era.drug_era_start_date
- and condition_era.condition_era_start_date - drug_era.drug_era_start_date <= 30
- ) sub1
- on sub1.drug_concept_id = drug_hoi_relationship.drug and sub1.condition_ concept_id = drug_hoi_relationship.hoi
- where evidence_type in
- ('MEDLINE_MeSH_CR','MEDLINE_MeSH_ClinTrial','MEDLINE_SemMedDB_CR','MEDLINE_SemMedDB_ClinTrial','SPL_EU_SPC')
- ) sub2
- group by rxnorm_drug, snomed_hoi, evidence_type, evidence_linkout
- order by pcount desc;

**-- RESULTS**

- *rxnorm_drug | snomed_hoi | evidence_type | evidence_linkout | pcount*
- Acetaminophen| Edema| MEDLINE_SemMedDB_CR| https://goo.gl/ikKucQ | 14
- Lisinopril| Abdominal pain| MEDLINE_MeSH_CR| https://goo.gl/49EvSE | 14
- Albuterol| Atrial fibrillation| MEDLINE_MeSH_CR| https://goo.gl/IVPdzx | 13
- Chlorthalidone| Hyperlipidemia| MEDLINE_MeSH_ClinTrial| https://goo.gl/gMTfzk | 13
- Metformin| Edema| MEDLINE_MeSH_ClinTrial| https://goo.gl/kL3GIz | 13
- Enalapril| Anemia| MEDLINE_MeSH_CR| https://goo.gl/AB1Lue |13
- Captopril| Anemia| MEDLINE_MeSH_CR| https://goo.gl/Cfhkzt | 13

### The system satisfies non-functional requirements

The new system is entirely open source so that any interested researcher can download, run, modify, and extend the code to fit their purposes. For example, the system currently does not have a data source that provides adverse event data from social media sources. Such new evidence sources could be integrated in the platform by developing additional Python ETL modules [42]. The system also provides systematic evidence to facilitate OHDSI’s methodological research efforts to enable the design, development and evaluation of new analytical approaches to observational research, and provides the basis for estimating systematic error and performing empirical calibration in all population-level estimation routines [43].

## Discussion

Drugs on the market need to be monitored for public safety. A safety physician or risk management analyst has to review all the available information for drug safety issues, following what is currently a highly manual, time-intensive, and error-prone process. Improvement of the automation of bringing all the relevant evidence together in a consumable format will help such individuals better achieve their goals. LAERTES provides an open source framework that uses OHDSI technology to bring together evidence from multiple sources in a way that will enable the development of software to meet the user goals mentioned at the beginning of this paper.

While the current version of LAERTES provides useful functionality, it also leaves opportunities for further research. In order to integrate the evidence sources, drug and HOI concepts have to be converted into RxNorm and SNOMED-CT concepts, respectively. Table 2 shows that there are many cases where this conversion is incomplete and some parts of the source data are not integrated. Future work will examine ways to improve the translation and mapping process.

Another opportunity for research is on how to appropriately aggregate evidence at different levels of a hierarchy of HOI concepts. For example, we have observed that some evidence sources map the concept myocardial infarction (concept identifier 4329847) directly to the SNOMED equivalent, whereas others map directly to a more specific concept like acute myocardial infarction (concept identifier 312327). The OMOP Vocabularies can be used to address this issue using the hierarchy provided. However, unlike drugs, it is not always clear the appropriate level to rollup HOI concepts. Future work will examine this issue in more detail and explore the use of alternate definitions of concept similarity [44].

### Limitations

Evidence sources that do not use standardized terminologies have to be processed to map the source concept names to SNOMED-CT and RxNorm concept codes. Even for evidence sources that use controlled terminologies, there is often a conversion process required to integrate them with all of the included LAERTES sources. One limitation is that, because of the prototype nature of the current version of LAERTES, we currently do not have precise precision/recall metrics for each of the methods we used (Table 2).

Another limitation is that some terminologies are incompatible and result in imperfect mappings. In cases where mapping is incomplete, some parts of the source data will not be integrated. We mentioned above the example of the MEDLINE source using MeSH to code drugs at the ingredient level, while drugs in US product labels are coded at the clinical drug level. These cases can be addressed with the drug rollup queries described above. Table 3 represents overlap between the general categories of sources at the drug ingredient level using the drug concept rollup strategy. However, unlike drugs, the appropriate level to rollup HOI concepts is not always clear. Some examples seem straightforward such as the myocardial infarction example mentioned above which can be addressed using ancestor/descendant relationships in the OMOP vocabulary. However, it is less clear how to apply the strategy to the universe of HOI concepts because there exists many different hierarchies depending on the disease and the level of detail present in SNOMED. We did not attempt to address this issue in the LAERTES prototype.

## Conclusion

Post-marketing drug safety surveillance is an important, continuous, and demanding process. Accurate and timely identification and verification of safety signals remains a major challenge. Drug–HOI evidence exists in many sources, which are disjointed, and variable in their representation of drugs and HOIs. The current practice of reviewing drug-HOI evidence is a highly manual time intensive process that is wrought with opportunity for failure. Improvement of the automation of bringing this information together in a consumable format will greatly improve the pharmacovigilance field. LAERTES provides a framework that accepts data from multiple sources and leverages the OMOP Vocabulary to translate those sources to one terminology for drugs and one for conditions, while also enabling integration with clinical data stored in the OMOP CDM. In addition, LAERTES is open-source facilitating domain experts’ participation in its development. As the breadth of evidence available on drug-HOI associationsis too wide for any individual to be expert, an open-source model allows for niche domain experts to contribute their knowledge improving the usefulness of LAERTES for the entire drug safety community.

This paper started with a motivating safety physician and risk management analyst user story to help guide LAERTES use cases. LAERTES has already collated several of the evidence sources individuals in this role would traditionally use for investigating drug-HOI signals. The LAERTES workgroup believes that the framework is in place to address the motivating example but realizes there is more work to be done. The workgroup fully expects and welcomes feedback from the community; for example, on new data sources, improvements to the Web API, and user interface design.

## Abbreviations

ADE: Adverse drug event
AE: Adverse event
API: Application programming interface
CDM: Common data model
CTD: Comparative Toxicogenomics Database
DB: Database
DBMS: Database management system
EBGM: Empirical Bayes Geometric Mean
EHR: Electronic health record
ETL: Extract, translate, and load
EU: European Union
FAERS: Food and Drug Administration Adverse Event Reporting System
FDB: First data bank (R)
HOIs: Health outcomes of interest
IC: Information component
LAERTES: Large-scale adverse effects related to treatment evidence standardization
MDRR: Minimal detectable reporting ratio
MedDRA: Medical dictionary for regulatory activities
MeSH: Medical subject headings
NLP: Natural language processing
OA: Open annotation data model
OHDSI: Observational Health Data Sciences and Informatics
OMOP: Observational Medical Outcomes Partnership
PPV: Positive predictive value
RDF: Resource Description Framework
REST: Representational state transfer
SNOMED-CT: Systematized Nomenclature of Medicine - Clinical Terms
URIs: Uniform Resource Identifiers
US: United States
W3C: World Wide Web Consortium
